# The American Academy of Medicine

**Published:** 1885-12

**Authors:** 


THE AMERICAN ACADEMY OF MEDICINE.

The ninth annual meeting of the American Academy of Medicine was held in New York, October 28th and 29th.

The following papers were presented October 28th:

"The Study of Medicine as a Means of Education." By ROBERT LOWRY SIBBERT, A. M., M. D., of Carlisle, Pa.

"Medical Supervision in Student Life." By CHARLES MCINTIRE, JR., A. M., M. D., of Easton, Pa.

"Western North Carolina as a Health Resort." By HENRY O. MARCY, A. M., M. D., of Boston, Mass.

"The Importance of Climatology Considered as a Regular Branch of Study in Medical Colleges." By E. H. M. SELL, A. M., M. D., of New York.

At eight o'clock, P. M., Address, by ALBERT L. GIHON, A. M., M. D., Medical Director, United States Navy, President of the Academy:—"What is Medicine?"

After which the annual collation was participated in by the members.

During the second day's session the following papers were presented:

"Medical Evidence." By THOMAS J. TURNER, A. M., M. D., PH. D., Medical Director United States Navy.

"Report on Laws Regulating the Practice of Medicine in the United States and Canada. By RICHARD J. DUNGLISON, A. M., M. D., of Philadelphia, Pa., and HENRY O. MARCY, A. M., M. D., of Boston, Mass.

"Health Officers, Ancient and Modern." By BENJAMIN LEE, A. M., M. D., Secretary of the State Board of Health of Pennsylvania.

"Micro-organisms and their Relation to Disease." By SAMUEL N. NELSON, A. B., M. D., of Cambridge, Mass.

"Observations on the Relation of Bacteria to Certain Puerperal Inflammations." By ERNEST W. CUSHING, A. B., M. D., of Boston, Mass.

"Medical Licenses and Medical Honors." By EDWARD JACKSON, A. M., M. D., of Philadelphia, Pa.

"The Physician and his Patient." By JOHN DEVIN KELLY, A. M., M. D., of Utica, N. Y.

"Sketches of some of the Original Members of the Delaware State Medical Society." By LEWIS P. BUSH, A. M., M. D., of Wilmington, Delaware.

In his address the President of the Academy advocated an extension of the conditions of membership in the Academy, in the belief that its usefulness will be still further increased, The matter will be considered at the next annual meeting.

Relative to preliminary education for medical students, which has been one of the important objects for which the Academy was organized, the following resolution was adopted:

*Resolved*, That a committee of three be appointed by the President, to report at the next annual meeting, instructed to prepare a statement of the best preliminary education for medical students, and also a statement of the minimum attainment which medical schools should require of students before admitting them to the study of medicine.

It was also resolved that a committee of two be appointed, "whose duty it shall be to report on the requirements as to preliminary education of the various medical colleges in the United States and in Canada." The newly elected members of the Academy are:

Drs. S. M. Nelson, Cambridge, Mass; J. H. W. Chestnut, Philadelphia; Rufus W. Bishop, Chicago; E. E. Mariott, Springfield, Mass.; George N. Acker, Washington, D. C.; H. V. Logan, Scranton, Pa.; H. W. Elmer, Bridgton, N. Y.; H. F. Hansell, Philadelphia; E. W. Cushing, Boston, Mass.; J. S. Wight, Brooklyn, N. Y.; William Osgood, North Yarmouth, Me.; C. A. Packard, Bath, Me.; W. K. Oake, Aubern, Me.; A. Mitchel, Brunswick, Me.; D. A. Robinson, Bangar, Me.; C. A. Ring, Portland, Me.; J. A. Spaulding, Portland, Me.; G. W. Marshall, Milford, Del.; Wm. A. Hugie, Charleston, S. C.; J. B. Shapley, St. Louis, Mo.; M. H. Post, St. Louis, Mo.; C. E. Briggs, St. Louis, Mo.; J. S. Alleymen, St.

Louis, Mo.; N. S. Davis, Jr., Chicago, Ill., and Charles Edgar Cook, Mendota, Illinois. Dr. Henry H. Smith and Dr. S. Weir Mitchell, of Philadelphia were elected to honorary membership.

The following were elected as the officers for the ensuing year:

President—Dr. R. S. Sutton, of Pennsylvania.

Vice-Presidents—Drs. L. P. Bush, of Delaware; S. J. Jones, of Illinois; R. L. Sibbett, of Pennsylvania and F. H. Gerish, of Maine.

Secretary and Treasurer—Dr. R. J. Dunglison, of Pennsylvania.

Assistant Secretary—Dr. C. McIntyre, of Pennsylvania.

Pittsburg, Pa., was selected as the place for the next annual meeting, on the third Tuesday in September, 1886. The Academy then adjourned.

